# MicroRNAs miR-146a/b negatively modulate the senescence-associated inflammatory mediators IL-6 and IL-8

**DOI:** 10.18632/aging.100042

**Published:** 2009-04-21

**Authors:** Dipa Bhaumik, Gary K. Scott, Shiruyeh Schokrpur, Christopher K. Patil, Arturo V. Orjalo, Francis Rodier, Gordon J. Lithgow, Judith Campisi

**Affiliations:** ^1^Buck Institute for Age Research, Novato, CA 94945, USA; ^2^Lawrence Berkeley National Laboratory, Berkeley, CA 94720, USA; ^3^Present Address: University of California at Los Angeles, Los Angeles CA 90095, USA

**Keywords:** miRNA, DNA damage, IL-1α, IL-6, IL-8, inflammation

## Abstract

Senescence is a cellular program that irreversibly arrests the proliferation of damaged cells and induces the secretion of the inflammatory mediators IL- 6 and IL-8 which are part of a larger senescence associated secretory phenotype (SASP).  We screened quiescent and senescent human fibroblasts for differentially expressed microRNAS (miRNAs) and found that miRNAs 146a and 146b (miR-146a/b) were significantly elevated during senescence.  We suggest that delayed miR-146a/b induction might be a compensatory response to restrain inflammation.  Indeed, ectopic expression of miR-146a/b in primary human fibroblasts suppressed IL-6 and IL-8 secretion and downregulated IRAK1, a crucial component of the IL-1 receptor signal transduction pathway.  Cells undergoing senescence without induction of a robust SASP did not express miR-146a/b.  Further, IL-1α neutralizing antibodies abolished both miR-146a/b expression and IL-6 secretion.  Our findings expand the biological contexts in which miRNA-146a/b modulates inflammatory responses.  They suggest that IL-1 receptor signaling initiates both miR-146a/b upregulation and cytokine secretion, and that miR-146a/b is expressed in response to rising inflammatory cytokine levels as part of a negative feedback loop that restrains excessive SASP activity.

## Introduction

Cellular senescence is a cell fate program triggered by potentially oncogenic stimuli and stresses that prevent aged or abnormal cells from further proliferation [1,2].  Several stimuli, including repeated proliferation, growth stimulation coordinated with cell-cycle arrest, DNA damage and expression of activated oncogenes cause mammalian cells to enter into the essentially irreversible growth senescent arrest and acquire the morphological and behavioral features of senescent cells  [3-5].

Senescent cells have been shown to accumulate in a variety of  aging tissues as well as several premalignant and malignant lesions [1].  Because cellular senescence eliminates the proliferative capacity of damaged cells it is a potent tumor suppressing mechanism [1,6].  However senescence also prevents the replacement of cells lost owing to age, injury or apoptosis.  Thus, the senescence response is likely a tradeoff between tumor suppression and tissue regeneration.  Senescence may therefore be considered an example of evolutionary antagonistic pleiotropy, whereby a trait that confers a selective advantage early in life (tumor suppression) may be retained even though it also has deleterious effects later in life [7].

Senescent human cells exhibit numerous changes in gene expression, many of which relate to the growth arrest [8].  Senescent cells also develop a senescence-associated secretory phenotype (SASP) [9].  The SASP is characterized by the secretion of a wide range of growth factors, cytokines, extracellular matrix proteins and degradative enzymes, most of which can alter the local tissue microenvironment [9-13].  The SASP is controlled in a modular fashion:  for example, the DNA damage response kinase ATM is required for the upregulation of some, but not all, SASP factors [14].  Of particular interest SASP is characterized by high level secretion of the cytokines, IL-6 and IL-8, which are key mediators of inflammation.  Inflammation is important for development of cancer as well as many other age-related diseases [15]. Furthermore, IL-6 and IL-8 were recently shown to reinforce the senescent growth arrest [15-17].  Thus, understanding the mechanisms that regulate IL-6 and IL-8 in association with senescence is important for understanding biological processes as diverse as tumor suppression and the development of age-related diseases, including cancer.

Recent studies have identified microRNAs (miRNAs) as important regulators of diverse biological processes.  miRNAs are ~22 nucleotide non-coding regulatory RNAs that are evolutionary conserved from nematodes to humans [18,19]. Primary miRNAs are initially transcribed by RNA polymerase II as larger precursors, which are then cleaved by a nuclear complex containing the ribonuclease Drosha and DCGR8.  The cleaved product is a hairpin RNA ~65 nucleotides in length known as the pre-miRNA [20].  The pre-miRNA is further processed to the mature miRNA by the cytosolic enzyme Dicer.  The mature miRNA is then incorporated into the RNA-induced silencing complex (RISC). The miRNA-RISC complex binds to target messenger RNAs (mRNAs), often in the 3' untranslated regions, and either promotes mRNA degradation or translational repression [21-23].  Each miRNA has the potential to regulate the expression of multiple mRNA targets.

miRNAs regulate a broad range of phenotypes including embryonic development, cell proliferation, differentiation and apoptosis [24-27].   miRNAs also control various activities of the immune system [28-30].  Recent studies show that miRNAs are important etiological or facilitating factors in the pathogenesis of several diseases, including cancer, diabetes, rheumatoid arthritis, and Alzheimer's disease [31-35].

miRNAs have also been implicated in the control of aging and cellular senescence.  Mutation of miR-lin-4 in *C.elegans *dramatically shortens life span [36]. Additionally members of the miR-34 family of miRNAs were recently shown to suppress cell proliferation and be direct targets of the p53 tumor suppressor protein which is required for the senescence growth arrest [31,37]. Indeed, overexpression of miR-34a in normal human IMR90 fibroblast caused a senescence growth arrest [37].  Similarly the MDM2 inhibitor Nutlin3A induced miR-34 and senescence in human fibroblasts via activation of p53 [38].   In mouse embryonic fibroblasts (MEFs), miR-20a induced senescence, in this case by upregulating the p16INK4A tumor suppressor protein [39].  Finally ablation of Dicer in MEF's induced senescence by upregulating p53, indicating that miRNAs play both positive and negative roles in regulating the senescence arrest [40].  In contrast to a rising understanding of how miRNAs modulate the senescence growth arrest, virtually nothing is known about whether or how miRNAs regulate any component of the SASP.

Here we report, that the levels of two related miRNAs, miR-146a and 146b (miR-146a/b), increase in senescent human fibroblasts in an interleukin IL1α dependent manner, but only when high levels of IL-6 and IL-8 secretion accompany senescence.  In the context of the SASP, we propose that increased expression of miR-146a/b serves to restrain excessive secretion of the inflammatory cytokines IL-6 and IL-8, thereby limiting senescence-associated inflammation.

## Results

### Induction of miR-146a and miR-146b by senescent human HCA2 fibroblasts

**Figure 1.**
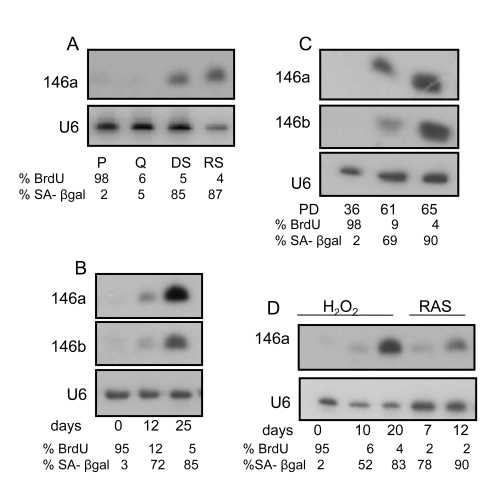
miR146a/b expression increases in senescent HCA2 fibroblasts. (**A**) Northern blot analysis of total RNA prepared from proliferating (P), quiescent (Q), damage (bleomycin)-induced senescent (DS) and replicatively senescent (RS) HCA2 cells.  We analyzed 10 μg of RNA from P, Q and DS cells, but 5 μg of RNA from RS cells.  After separation and transfer to membranes, the blots were probed for miR-146a.  Equal RNA loading was confirmed by probing for the small RNA species U6.  Values for the percentage of cells incorporating bromodeoxyuridine (% BrdU) or expressing the sensecence-associated beta-galactosidase (% SA-β-gal) are indicated below each lane.  (**B**)  Northern blot analysis of RNA from DS cells. Cells were harvested for RNA at the days indicated after cells were induced to senesce by bleomycin.  The blot was initially probed for miR-146a, then stripped and reprobed for miR-146b.  The proliferation levels (% BrdU) and % cells that express the SA-β-gal are indicated.  (**C**)  Northern blot analysis of replicatively senescencing cells.  Cells were harvested at the PD (population doubling level) indicated below the figure.   The proliferation levels (% BrdU) and % cells that express the SA-β-gal are indicated.  (**D**)  Northern blot analysis of cells treated with H_2_O_2_ (0.1 mM for 2 h) or infected with the lentivirus expressing oncogenic RASV12.  Cells were harvested for RNA at the indicated days after treatment.  The proliferation levels (% BrdU) and % cells that express the SA-β-gal are indicated.

In screening arrays of known miRNAS for those that are differentially expressed by quiescent versus senescent cells (C. Patil, manuscript in preparation), we found that miR-146a and miR-146b were expressed at significantly higher levels by senescent HCA2 cells, which are normal human fibroblasts from neonatal foreskin.  This was true whether senescence was induced by a DNA damaging agent (bleomycin, which causes DNA double strand breaks) (DS) or replicative exhaustion (RS).  We validated the array results for miR-146a expression by northern analysis.  miR-146a was readily detectable in senescent cells, but was undetectable in proliferating (P) or quiescent (Q) cells (Figure 1A).  In these and subsequent samples, senescence was confirmed by the low percentage of proliferating (BrdU-incorporating) cells and high percentage of cells that stained positive for the senescence associated β-galactosidase (SA-β-gal) (Figure 1; Experimental Procedures). Because quiescent cells, which are not senescent but rather temporarily growth arrested, expressed low to undetectable levels of miR-146a, as did proliferating presenescent cells, we conclude that robust expression of miR-146a is specifically associated with senescence and not simply with growth arrest.  In the experiments that follow, we used proliferating cells as a negative control for miR-146a/b expression.

We investigated the kinetics of induction of miR-146a/b expression following induction of senescence by different stimuli.  Following a senescence-inducing dose of the DNA damaging agent bleomycin, miR-146a/b was first detected approximately 12 days later (Figure 1B).  By comparison, the SASP is first evident 3-4 days after a senescence-inducing dose of DNA damage, and completely established within 5-7 days after senescence induction [9,14]  Thus, the levels of miRNA-146a/b remained undetectable or very low during the interval in which the SASP developed in response to DNA damage (not shown), and were detected by northern analysis only several days later.  By 25 days after senescence was induced by DNA damage, the levels of miRNA-146a/b were maximal (Figure 1B).  At this time point, the few cells that were able to repair the damage and resume growth comprised only a small fraction of the population and thus the population remained largely senescent with ~5% BrdU incorporation and 85% SA-β-gal activity.  We obtained similar results when we analyzed replicatively senescent cells (Figure 1C).  At PD 65, when the cells were nearly completely senescent (4% BrdU incorporation, 90% positive for SA-β-gal), miR-146a/b expression was higher than at PD 61, when the cells were less completely senescent ( 5% BrdU incorporation, 69% SA-β-gal positive cells).  Further, miR-146a/b followed a similar expression pattern when we induced senescence by oxidative stress (hydrogen peroxide treatment) (Figure 1D, left lanes) or the oncogene RASV12 (to cause oncogene-induced senescence) (Figure 1D, right lanes).  Across all conditions tested, miRNA-146a/b expression remained low during the early period of senescence, when other phenotypes (growth arrest, SA-β-gal expression, and the SASP) were well underway, but rose to higher levels during a later period after these senescence-associated phenotypes had been fully established.

### miR-146a/b suppresses IRAK-1 expression and reduces IL-6 and IL-8 secretion in HCA2 fibroblasts

**Figure 2.**
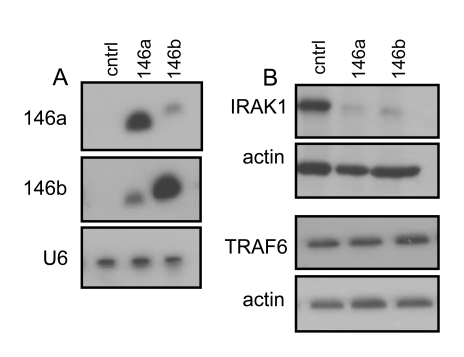
IRAK1 but not TRAF6 levels are reduced in HCA2 cells overexpressing miR146a and miR 146b. (**A**) Northern blot analysis of total RNA prepared from control (insertless virus-infected) HCA2 cells (cntrl), cells infected with a miR-146a-expressing virus (146a) and cells infected with a miR-146b-expressing virus (146b).  8 μg of total RNA was loaded in each lane.   (**B**)  Western blot analysis of total protein lysates prepared from proliferating cells (cntrl, PD32), or cells overexpressing miR-146a or miR-146b, and analyzed for IRAK1 (top panel) and TRAF6 (bottom panel). Actin protein levels served as a loading control.

To determine the role of miR-146a/b, in the phenotypes of human fibroblast, we stably infected proliferating cells with either a control lentivirus or lentiviruses expressing miR-146a or miR-146b (Figure 2A).  The miR-146a/b overexpressing fibroblasts displayed no obvious morphological alterations and maintained a proliferation rate comparable to that of control cells (data not shown).  Thus, miR-146a/b did not induce a quiescence or senescence growth arrest.

As discussed above, senescent cells robustly secrete the inflammatory cytokines IL-6 and IL-8.  Recent reports identified miR-146a/b as negative regulators of inflammatory cytokine expression during immune reactions and cancer cell invasiveness [9,41,42].  We therefore asked, whether miR-146a/b modulated the secretion of inflammatory cytokines by senescent cells.  First, we determined the effect of miR-146a/b over-expression on IRAK1 and TRAF-6.  These proteins are established miR-146a/b targets and key downstream components of the IL-1 and Toll-like receptor signaling cascades, which ultimately regulate the expression of inflammatory cytokines such as IL-6 and IL-8 [41,42].  HCA2 fibroblasts that overexpressed miR-146a/b had markedly reduced levels of IRAK1 (Figure 2B).  However, the levels of TRAF6 remained unaltered in these cells (Figure 2B).  Thus, at least in HCA2 cells, miR-146a/b targeted IRAK1, but not TRAF-6.

**Figure 3.**
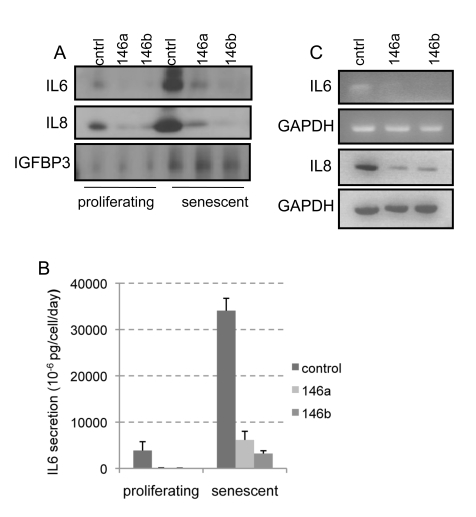
Overexpression of miR-146a/b suppresses basal and senescence-associated secretion of IL-6 and IL-8 in HCA2 cells. (**A**) Western blot analyses of TCA-precipitated proteins prepared from CM collected over 24 h from cells infected with the lentivirus backbone (cntrl) or lentiviruses expressing miR-146a (146a) or miR-146b (146b).  The blot was analyzed for IL-6, IL-8 and IGFBP3.  Equal loading was based on cell number prior to collection of CM and IGFBP3 levels.  Proliferating indicates cells described in Figure 2A.  The same cells were treated with bleomycin and CM was harvested 11 days later (senescent). (**B**)  IL-6 in CM from the cell populations described in Fig 3A was measured by ELISA.  The data are reported as 10^-6^ pg per cell per day. (**C**)  RT-PCR analysis of transcript levels of IL-6 and IL-8 in miR-146a/b-overexpressing cells.  RNA collected from proliferating cells was used as the control (cntrl).

Because overexpression of miR-146a/b in human MDA-MB-231 breast cancer cells, reduced the levels of secreted IL-6 and IL-8 [41] and since IRAK1 is a key mediator of the expression of IL-6 and IL-8, we compared the basal levels of secreted IL6 and IL8 in control and miR-146a/b overexpressing HCA2 cells.  We collected conditioned medium (CM) from these cells over a 24 h period and assayed the CM for IL-6 and IL-8 by western analysis (Figure 3A, proliferating) and ELISA (Figure 3B, proliferating).   We observed a marked reduction in basal secretion of IL-6 and IL-8 in the miR-146a/b overexpressing cells relative to control cells.  We normalized these measurements, against cell number and, where applicable, against the level of secreted IGFBP3 (Figure 3A), a protein that is not influenced by miR-146a/b expression [41].

To determine whether miR-146a/b influenced the increased IL-6 and IL-8 secretion that accompanies the senescence, we induced the miR146a/b overexpressing HCA2 fibroblasts to senesce by DNA-damage (bleomycin).  Western and ELISA analyses of CM collected 11 days after DNA damage showed that IL-6 and IL-8 secretion by senescent miR-146a/b-overexpressing cells, was strikingly reduced compared to control senescent cells (Figure 3A-B right panel).  RT-PCR analysis (Figure 3C), showed that miR-146a/b reduced the levels of IL-6 and IL-8 transcripts, indicating that miR-146a/b exerts these effects by decreasing transcription or promoting mRNA degradation.  Together, these observations establish that miR146a/b expression is sufficient to negatively regulate IL-6 and IL-8 secretion in both pre-senescent and senescent human fibroblasts.

### miR-146a/b expression increases only in senescent human fibroblasts that have robust IL-6 secretion

**Figure 4.**
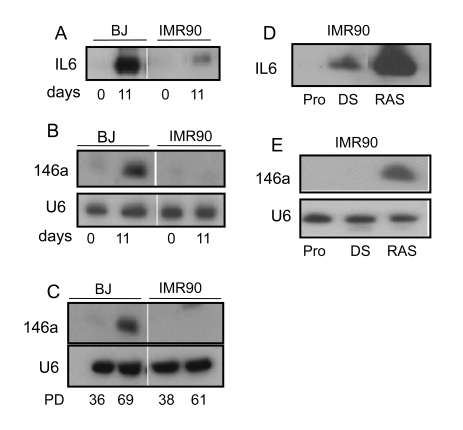
miR-146a/b increase in senescent fibroblasts that secrete high levels of inflammatory cytokines. (**A**) & (**B**)  Proliferating BJ (PD 36) and IMR90 (PD 38) cells were treated with bleomycin to induce senescence.  CM and RNA were harvested 11 d later. (**A**) Western analysis for secreted IL-6.  (**B**) Northern analysis for miR-146a. (**C**) Northern analysis for miR-146a levels in replicatively senescent BJ and IMR90 cells.  The PD levels at which cells were harvested for analysis is given below each lane.  BJ cells reach complete senescence after approximately 70 PDs, whereas IMR90 cells are nearly completely senescent by PD61.  (**D**) & (**E**) Proliferating IMR90 cells (PD40) were either untreated (Pro), treated with bleomycin (DS) or infected with the lentivirus expressing oncogenic RAS (RAS).  CM and RNA were collected 11 days after treatment or infection.  (**D**) Western analysis for IL-6 in CM.  (**E**) Northern analysis for miR-146a and U6 (control) levels.

To determine whether miR-146a/b negatively regulates IL-6 and IL-8 secretion in other human fibroblast strains, we examined BJ and IMR90 primary human fibroblasts.  BJ, like HCA2, fibroblasts are derived from neonatal foreskin, whereas IMR90 fibroblasts are derived from fetal lung.  Moreover, upon senescence, BJ and HCA2 cells express a robust SASP, whereas IMR90 cells express a less robust SASP, and, in particular, secrete less IL-6 and IL-8 [9].  We confirmed that senescent IMR90 cells secreted about 8-fold lower levels of IL-6 than senescent BJ cells when CM were analyzed 11 days after induction of senescence by DNA damage (bleomycin) (Figure 4A).  Notably, miR-146a levels were also substantially lower in senescent IMR90 compared to senescent BJ cells; in fact, mi-146a was essentially undetectable by northern analyses in senescent IMR90 cells but readily detectable in BJ cells (Figure 4B).  Additionally, replicatively senescent IMR90 cells also expressed undetectable levels of miR-146a, assayed by northern blotting, whereas miR-146a expression was easily detectable in near replicatively senescent BJ cells (Figure 4C).  Likewise, senescent cells of the strain WI-38, also derived from fetal lung and exhibiting the low SASP characteristics of IMR90 [9], did not express detectable levels of miR-146a/b (data not shown).

Because of the correlation between the magnitude of the inflammatory cytokine component of the SASP and miR-146a/b expression, we asked whether IMR90 might express higher levels of miR-146a under conditions that induced higher inflammatory cytokine secretion.  We previously showed that RAS oncogene-induced senescence results in a more robust SASP than damage-induced senescence [9].  We therefore measured the levels of IL-6 secretion (Figure 4D) and miR-146a/b expression (Figure 4E) in IMR90 cells induced to senesce by oncogenic RAS.  Oncogenic RAS significantly increased both IL-6 and miR-146a in IMR90 cells.  There was a close parallel between miR-146a/b expression and robustness of inflammatory cytokine secretion.

### IL1-α upregulates miR146a/b in senescent human fibroblasts

**Figure 5.**
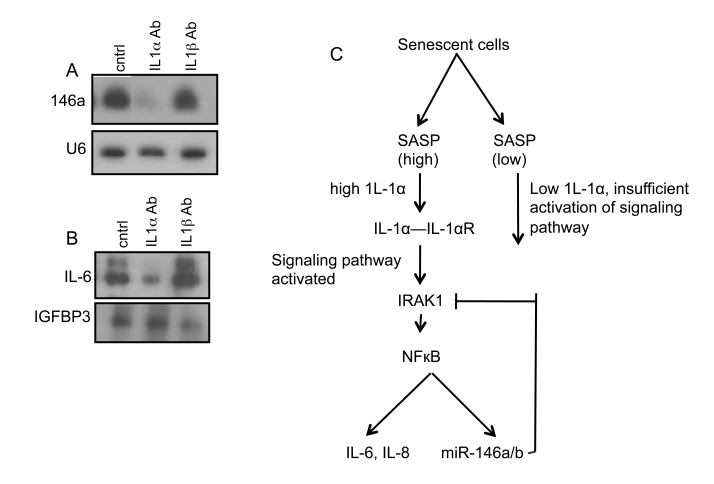
IL-1α upregulates miR-146a in senescent cells. (**A**) Northern analysis for miR-146a levels in damage-induced senescent HCA2 cells treated with neutralizing antibodies against IL1-α and IL1-β.  HCA2 cells (PD 35) were used and induced to senesce by treatment with bleomycin.  Cells were harvested for RNA 11 days later. Details of the procedure are described in ‘Experimental Procedures.  (**B)** Western analysis for IL-6 in damage-induced senescent HCA2 cells treated with neutralizing antibodies to IL-1α and IL-1β.  CM were harvested 11 days after bleomycin treatment. (**C**)  Model for the role of miR-146a/b in senescent cells: In response to a high SASP (right branch), IL-1α interacts with the IL-1α receptor (IL-1αR) and the signaling pathway that involves IRAK1 is fully activated.  This activation leads to the well-documented activation of the transcription factor NFкB and production of IL-6, IL-8 and also miRNA-146a/b. miRNA-146a/b is a component of a negative feedback loop and acts to downregulate the levels of IRAK1, hence restraining the levels of IL-6 and IL8.  However, in response to a low SASP (left branch), the signaling pathway is not sufficiently activated.  Thus there is a low level of IL-6 and IL-8 secretion and miRNA-146a/b is not upregulated.

To determine the mechanism that connects miR-146a/b expression and IL-6 and IL-8 secretion in human fibroblasts, we explored the role of IL-1 signaling which is a master regulator of inflammatory cytokine secretion.  Because IRAK1 is both a primary target of miR-146a/b and an essential downstream component of the IL-1 receptor signaling system, we tested the IL-1 receptor ligands, IL-1α and IL-1β, as strong candidates for ability to upregulate both miR-146a/b expression and IL-6 and IL-8 secretion.  IL-1β is a SASP component [9] and senescent cells contain high levels of membrane-bound IL-1α have also been observed in senescent cells (A. Orjalo, manuscript in preparation).  We added neutralizing antibodies against IL-1α or IL-1β to the culture medium of HCA2 cells one day after treatment with the DNA damaging agent bleomycin, and collected RNA samples 10 days later.  Northern analysis showed that neutralizing antibodies against IL- 1α not IL-1β suppressed the senescence-associated upregulation of miR-146a (Figure 5A).  In parallel, we assessed IL-6 secretion levels in CM obtained in the presence of neutralizing antibodies.  Again, neutralizing antibodies against IL-1α, but not IL-1β, suppressed the senescence-associated secretion of IL-6 (Figure 5B).  Similar suppression was seen in the levels of IL-8 (data not shown).

These findings suggest the following model (Figure 5C): When the SASP inflammatory secretion levels are low (right branch), IL-1α levels and IL-1R signaling are low.  However, when inflammatory cytokine secretion is high, (left branch), high IL-1R signaling activates the downstream kinase IRAK1, ultimately resulting in activation of NFкB.  This transcription factor stimulates expression of the SASP cytokines, IL-6 and IL-8, as well as miR-146a/b.  By targeting the IRAK1 mRNA, miR-146a/b creates a negative feedback loop that restrains IRAK1 signaling and limits senescence-associated cytokine production.

## Discussion

 The role of miRNAs in promoting diverse cellular programs such as stem cell maintenance, differentiation and apoptosis underscores their emerging importance as regulators of myriad biological processes [24-27].  Here, we demonstrate a role for miRNA146a/b in the cell non-autonomous effects of cellular senescence, a phenomenon linked to both cancer and aging.

Following multiple forms of senescence-inducing treatments, miR146 levels increase sharply in senescent HCA2 cells rising from nearly undetectable baseline levels in proliferating and quiescent cells.  miR-146a/b expression is associated with a wide range of normal and pathological biology.  These miRNAs have been implicated in the biology of monocytic immune cells responding to lipopolysaccharide stimulation, human lung alveolar epithelial cell line responding to IL-1β induction, brain tissue from patients with Alzheimer's disease and synovial tissue from patients with rheumatoid arthritis [34,35,43,44]. In the majority of these situations, miR-146a/b acts as a negative regulator of inflammatory pathways.  In the immune system, miR-146a/b upregulation provides negative feedback on the innate immune system by targeting IRAK1 and TRAF6 [44].  These proteins are key components of the IL-1 and Toll-like receptor signaling system, of which NFкB activation is a primary downstream effector.  In agreement with earlier observations on immune cells, our previous work demonstrated that miR-146a/b negatively regulates NFkB activity and the inflammatory pathway, in breast cancer cells [41,42].  In the context of senescence, the results presented here expand and corroborate the role of miR-146a/b as negative regulators of inflammation and, in particular, underscore its influence as a negative regulator of excessive IL-6 and IL-8 secretion, a hallmark of senescence.

HCA2 human fibroblasts overexpressing miR-146a/b exhibited markedly decreased levels of IRAK1 as well as IL-6 and IL-8, under both proliferating and senescent conditions.  IL-6 and IL-8 expression was suppressed at the mRNA level, most likely a consequence of diminished NFкB activation following miR-146a/b-mediated reduction in IRAK1 protein levels [41].   In the context of senescence, this regulation was a consequence of IL-1R signaling specifically activated by IL-1α but not IL-1β. This specificity, however, might be cell type-specific. In studies of lung alveolar epithelial cells by Perry *et al*, stimulation with IL-1β caused a rapid increase in miR-146a levels but this did not impact the protein levels of either IRAK1 or TRAF6 [43].  However, inhibitors of miR-146a did enhance the levels of the pro-inflammatory cytokines IL8 and RANTES [43].  These studies suggest that miRNA-146a can function as a negative regulator of inflammation at the level of translation suppression, independent of the IL-1 signaling pathway involving IRAK1.  In contrast, miR-146a/b downregulated IRAK1 protein levels in human fibroblasts, and thereby suppressed expression of IL-6 and IL-8, in agreement with a model whereby miR-146a/b responds to robust activation of the IL-1α signaling pathway and negatively regulates the IL-6 and IL-8 expression driven by that pathway (Figure 5C) [44].

We found that regulation of miR-146a/b and the IL-6 and IL-8 inflammatory components of the SASP requires signaling through the IL-1R pathway.  Neutralizing antibodies against IL-1α sharply decreased both IL-6 secretion and miR-146a production. In contrast, neutralizing antibodies against IL-1β did not suppress inflammatory cytokine secretion, suggesting that IL-1β secretion levels at senescence are not sufficient to produce a significant level of IL-1R signaling.  Taken together, these results suggest the model shown in Figure 5C.  In senescent cells, activation of the IL-1 receptor signaling pathway is dependent upon sufficiently high IL-1α levels.  In HCA2 cells, a wide range of conditions that induce senescence promote IL-1 receptor activation, whereas in IMR90 cells only oncogene-induced senescence results  in sufficiently intense SASP to promote IL-6 and miR-146 expression; senescent fibroblasts that exhibit lower levels of SASP secretion do not induce miR-146 (Figure 4).

Thus, miR-146a/b in senescent fibroblasts that express a robust SASP negatively regulates IRAK1 protein levels, thereby dampening the IL-1 receptor signaling pathway and hence the expression and secretion of inflammatory molecules such as IL-6 and IL-8.  This negative feedback loop would serve to limit the deleterious effects of the SASP on surrounding tissues.  This safeguard may be particularly important when the local concentration of SASP factors is high, for example when senescent cells accumulate in premalignant nevi [45] or are induced after exposure to chemotherapeutic agents [9].

## Experimental procedures

Cells. Early passage HCA2 human foreskin fibroblasts were obtained from J Smith (University of Texas, San Antonio).  Early passage BJ human foreskin fibroblasts and IMR90 fetal lung fibroblast were obtained from ATCC. Cells were cultured at 37° C in a 10% CO_2_ incubator in DMEM with 10% fetal bovine serum.  293FT packaging cells (Invitrogen) were used to generate lentivirus.  We defined presenescent (proliferative) cells, as having undergone fewer than 35 population doublings and having a 24 h BrdU labeling index of ~95%.  Subconfluent cells (1500-4000/cm^2^) were made quiescent by washing with serum-free medium and incubating in 0.2% serum for 4 d. Proliferating cells were made replicatively senescent by serial passage in 10% serum.  For damage induced senescence, cells were plated at a density of 40000/cm^2^.  Two days later the cells were treated with bleomycin 40 μg/ml for 2 h. On the 11^th^ day or indicated day following treatment, cells were stained for the senescence-associated β-gal (SA-β-gal) marker [46] and DNA synthesis was measured over a 24 h interval using a BrdU labeling kit ( Roche Diagnostics).  Cultures that had > 80% SA-β-gal positive cells and ≤ 4% BrdU positive cells were considered senescent.

Lentiviral constructs, viruses and infections. The lentiviral miR-146a/b expression vectors were constructed as described [41].  The expression of miR-146a/b was under control of the cytomegalovirus promoter. The lentivirus encoding oncogenic RAS V12 has been described [14].  24 h following lentiviral infection cells were placed under puromycin (1 μg/ml) selection for 4 d.

Antibodies. IRAK1 ( SC 5288 ), TRAF6 (SC 8409**)** and IGFBP3 (SC 9028) antibodies were obtained from Santacruz Biotechnology, USA.  IL-6 (AF-206-NA), IL-8 (MAB208), IL-1α (MAB200) and IL-1β (MAB601) was obtained from R&D systems.

Northern blots. Total RNA was extracted using Trizol (Invitrogen, Carlsbad, CA) and analyzed by northern blotting.  Briefly, RNA was separated on 15% TBE- urea polyacrylamide gels (Invitrogen, Carlsbad, CA) and transferred onto Hybond Plus membranes  (Amersham, Piscataway, NJ) as previously described [47].  The blots were probed with an antisense miR-146a DNA oligonucleotide, striped and reprobed with an antisense miR-146b DNA oligonucleotide.  Oligonucleotides were^32^P end-labeled.  RNA loading was confirmed by probing for the small RNA species, U6.  Unless mentioned otherwise 10 μg of RNA was loaded in each lane.

For Northern analysis of RNA harvested from cells treated with neutralizing antibodies, IL-1α (0.6 μg/ml) and IL-1β (0.8 μg/ml) antibodies were added to the medium one day after cells were induced to senescence by bleomycin.  Fresh medium was added 6 d later and the medium was replenished with another aliquot of the neutralizing antibody at the same concentration.  RNA was harvested 11 d after bleomycin treatment.

RT PCR. Total RNA was used for RT-PCR analysis of IL-6 and IL-8 transcripts.  Reverse transcription was done using Super Script II (Invitrogen, Carlsbad,USA).  Following reverse transcription the products were was amplified for 30 cycles using appropriate primers.

Western Blots. Total protein extracts were used for western analysis of IRAK1, TRAF6 and actin (loading control).  CM was prepared by washing cells 3 times in PBS and incubating the cells in 0.2% serum in DMEM medium.  CM was harvested 24 h latter and protein precipitated with 15% TCA overnight.  Loading was based on equal cell number and where appropriate IGFBP3 was used as a loading control. For treatment with neutralizing antibodies, IL-1α (0.6 μg/ml) and IL-1β (0.8 μg/ml) was added 24 h prior to collection of CM.

ELISA. CM was prepared as described above except cells were incubated for 24 h in serum free DMEM.  ELISA was performed using kits and procedures from R&D (IL-6- #D06050).
